# Molecular Basis of Renal Adaptation in a Murine Model of Congenital Obstructive Nephropathy

**DOI:** 10.1371/journal.pone.0072762

**Published:** 2013-09-04

**Authors:** Brian Becknell, Ashley R. Carpenter, Jordan L. Allen, Michael E. Wilhide, Susan E. Ingraham, David S. Hains, Kirk M. McHugh

**Affiliations:** 1 Department of Pediatrics, College of Medicine, The Ohio State University, Columbus, Ohio, United States of America; 2 Division of Pediatric Nephrology, Nationwide Children’s Hospital, Columbus, Ohio, United States of America; 3 Center for Molecular and Human Genetics, The Research Institute, Nationwide Children’s Hospital, Columbus, Ohio, United States of America; 4 Biomedical Sciences Graduate Program, College of Medicine, The Ohio State University, Columbus, Ohio, United States of America; 5 College of Medicine, The Ohio State University, Columbus, Ohio, United States of America; 6 Center for Clinical and Translational Research, The Research Institute, Nationwide Children’s Hospital, Columbus, Ohio, United States of America; UCL Institute of Child Health, United Kingdom

## Abstract

Congenital obstructive nephropathy is a common cause of chronic kidney disease and a leading indication for renal transplant in children. The cellular and molecular responses of the kidney to congenital obstruction are incompletely characterized. In this study, we evaluated global transcription in kidneys with graded hydronephrosis in the *megabladder* (*mgb* ^−/−^) mouse to better understand the pathophysiology of congenital obstructive nephropathy. Three primary pathways associated with kidney remodeling/repair were induced in *mgb* ^−/−^ kidneys independent of the degree of hydronephrosis. These pathways included retinoid signaling, steroid hormone metabolism, and renal response to injury. Urothelial proliferation and the expression of genes with roles in the integrity and maintenance of the renal urothelium were selectively increased in *mgb* ^−/−^ kidneys. *Ngal/Lcn2*, a marker of acute kidney injury, was elevated in 36% of kidneys with higher grades of hydronephrosis. Evaluation of *Ngal^high^* versus *Ngal^low^* kidneys identified the expression of several novel candidate markers of renal injury. This study indicates that the development of progressive hydronephrosis in *mgb* ^−/−^ mice results in renal adaptation that includes significant changes in the morphology and potential functionality of the renal urothelium. These observations will permit the development of novel biomarkers and therapeutic approaches to progressive renal injury in the context of congenital obstruction.

## Introduction

Congenital obstructive nephropathy (CON) is a leading cause of chronic kidney disease (CKD) and end stage renal disease (ESRD) in children, particularly in boys with prune belly syndrome and posterior urethral valves [1]. The best available biomarker of residual renal function in children with CON is serum creatinine, even though it does not permit physicians to reliably predict which children with obstruction will develop significant CKD [2], [3]. Clearly additional studies are needed to clarify the cellular and molecular responses to CON in order to develop reliable diagnostic/prognostic markers of renal injury and therapeutic strategies to reduce the impact of CKD.

A significant body of work describes the renal response to acute obstruction principally through the use of partial or complete ureteral obstruction in large animal and rodent models [4]. In contrast, far less is known about the renal response to graded degrees of congenital hydronephrosis, a fact that is critical in understanding the pathophysiology of CON. To this end, we have developed the megabladder (*mgb* ^−/−^) mouse model of CON. *Mgb* ^−/−^ embryos develop an atonic bladder and exhibit hydronephrosis *in utero*, which persists postnatally and is exacerbated in males at sexual maturation [5]. Male *mgb* ^−/−^ animals eventually develop severe bilateral hydronephrosis resulting in death around six weeks of age from renal failure and severe dehydration [6].

Hydronephrosis in *mgb^−/−^* mice progresses at variable rates, such that by 3–4 weeks of age, a spectrum of obstruction occurs associated with mild to severe reductions in renal parenchyma and function [6]. This variability parallels that seen in humans with obstructive uropathy [1]. In this study, we utilized this variability to evaluate the global transcriptomes of *mgb* ^−/−^ versus control kidneys at varying grades of hydronephrosis. We hypothesized that distinct cellular responses would be observed in mild, moderate and severe hydronephrotic kidneys providing insight into the key molecular pathways responsible for the pathophysiology of CON as well as the identification of novel biomarkers for the staging of renal injury.

## Materials and Methods

### Ethics Statement

The following studies were conducted at The Research Institute at Nationwide Children’s Hospital under the Public Health Services approved Animal Welfare Assurance number A3544-01. All studies were approved by the Institutional Animal Care and Use Committee under protocol 02105AR.

### Animals

Mice were maintained according to the NIH Guide for the Care and Use of Laboratory Animals as previously described [5]. Age-matched, 23–30 day old, control and *mgb^−/−^* male mice were subject to renal ultrasound to establish the degree of hydronephrosis as previously published [7]. Of note, our method of grading hydronephrosis is based on the degree of parenchymal preservation, and does not directly correlate with clinical grading of hydronephrosis, such as the Society of Fetal Urology Grading System (http://www.sfu-urology.org/sfu_hydrone_grading.cfm). Kidneys with ≥67% parenchyma were considered mildly, 34–66% moderately and ≤33% severely affected. Mice were euthanized, kidneys extracted, snap frozen and stored at −80°C.

### RNA Extraction and Microarray Hybridization

Total RNA was extracted using mirVana™ kit (Life Technologies, Carlsbad, CA). RNA integrity was analyzed using Agilent 2100 Bioanalyzer Lab-On-A-Chip 6000 Series II chip (Agilent Technologies, Santa Clara, CA). Samples were hybridized to Agilent SurePrint G3 Mouse GE 8×60 K Microarray and scanned using Agilent G2505C Microarray Scanner. Raw data were quality-assessed, filtered for outliers and normalized to remove non-biological variation. Differential gene expression was defined using a 10% false discovery rate (FDR) and adjusted *p*-value <0.05 (Bioconductor LIMMA package). Data have been deposited in Gene Expression Omnibus (GEO accession: GSE48041).

### Pathway Analysis

Ingenuity Pathway Analysis (IPA; Ingenuity Systems, Redwood City, CA. http://www.ingenuitypathway.org; version 14197757) was used to evaluate the biological significance of co-regulated transcripts. mRNA with ≥2 fold change in expression, adjusted *p*<0.05, and FDR <10 were considered significant.

### qPCR

cDNA was prepared from kidney total RNA using random hexamers and reverse transcriptase. Triplicate PCR reactions were performed using 25 ng of cDNA as a template in 20 µL reactions containing 2X master mix and gene-specific primers/probes (Applied Biosystems, Foster City, CA). Simultaneous triplicate reactions included *Gapdh* as an endogenous control. Results were expressed using the 2^−ΔΔCT^ method by normalizing to a common pool of control kidney cDNA [8]. The average fold change ± standard error was graphed, and *p*-values were calculated based on comparison of individual fold change values in an unpaired two-tailed Student’s *t* test.

### Immunohistochemistry (IHC)

Formalin fixed, paraffin embedded kidneys were sectioned at 4 µm. Deparaffinized sections were rehydrated, subjected to antigen retrieval, peroxidase block, biotin block, and Superblock (Scytek, Logan, UT). Primary antibodies were incubated for 1 hour at the following dilutions: anti-Upk3a (Research Diagnostics Inc., Flanders, NJ.) 1∶500; anti-Krt14 (Covance, Princeton, NJ) 1∶1600; anti-Ki-67 (Abcam, Cambridge, MA) 1∶800. Biotinylated secondary antibody and HRP-conjugated streptavidin were implemented (Scytek). Slides were developed using diaminobenzamide (MP Biomedicals, Santa Ana, CA), counterstained with hematoxylin and visualized using an Olympus BX-51 microscope (Olympus, America, Center Valley, PA).

### Ki-67 Quantification

Ki-67 positive and Ki-67 negative nuclei within the renal urothelium were identified using anti-Ki-67 labeled and hematoxylin counterstained tissue sections. Longitudinally oriented, four-micron thick kidney sections were collected near the hilum. Briefly, contours were traced onto morphometric tissues of interest; kidney (red), renal urothelium (not shown), renal lumen (not shown) and large vasculature (blue). Individual Ki-67 positive (yellow crosshair) and Ki-67 negative (white dot) nuclei within the entirety of the renal urothelium contour were digitally marked. Graphic representations were rendered using StereoInvestigator software and quantitative analysis was obtained using Neurolucida Explorer software (MBF Bioscience, Williston, VT). Representative images from each experimental group are shown.

## Results

Global transcriptome analysis of 18 male *mgb^−/−^* kidneys with varying degrees of hydronephrosis and six age-matched male control kidneys identified 202 and 207 genes with ≥2-fold higher or lower expression in *mgb* ^−/−^ kidneys, respectively (Figure 1, Table S1). Differential expression of 70 target genes was validated by qPCR (Figures S1–S9).

**Figure 1:**
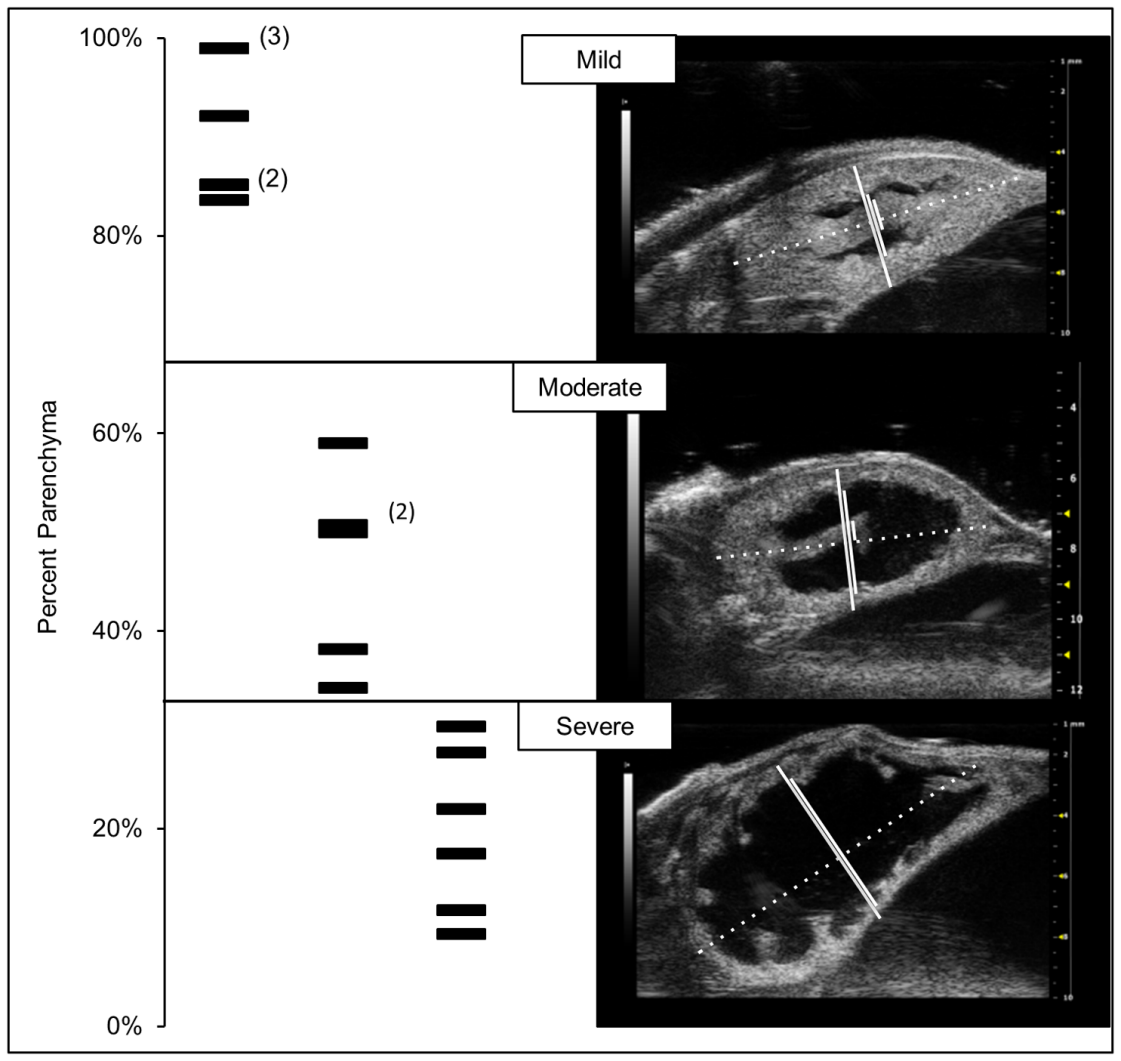
Ultrasound grading of hydronephrosis in *mgb^−/−^* mice. Left panel: percent parenchyma of *mgb^−/−^* kidneys undergoing transcriptome analysis. Black horizontal lines indicate individual kidney measurements. Right panel: representative sonograms of mild, moderate and severely hydronephrotic *mgb^−/−^* kidneys. Dotted white line indicates longitudinal renal length. Solid white lines from right to left indicate transverse renal width, renal pelvis diameter and renal papillae width with no measureable renal papillae width in the severe kidney.

**Figure 2:**
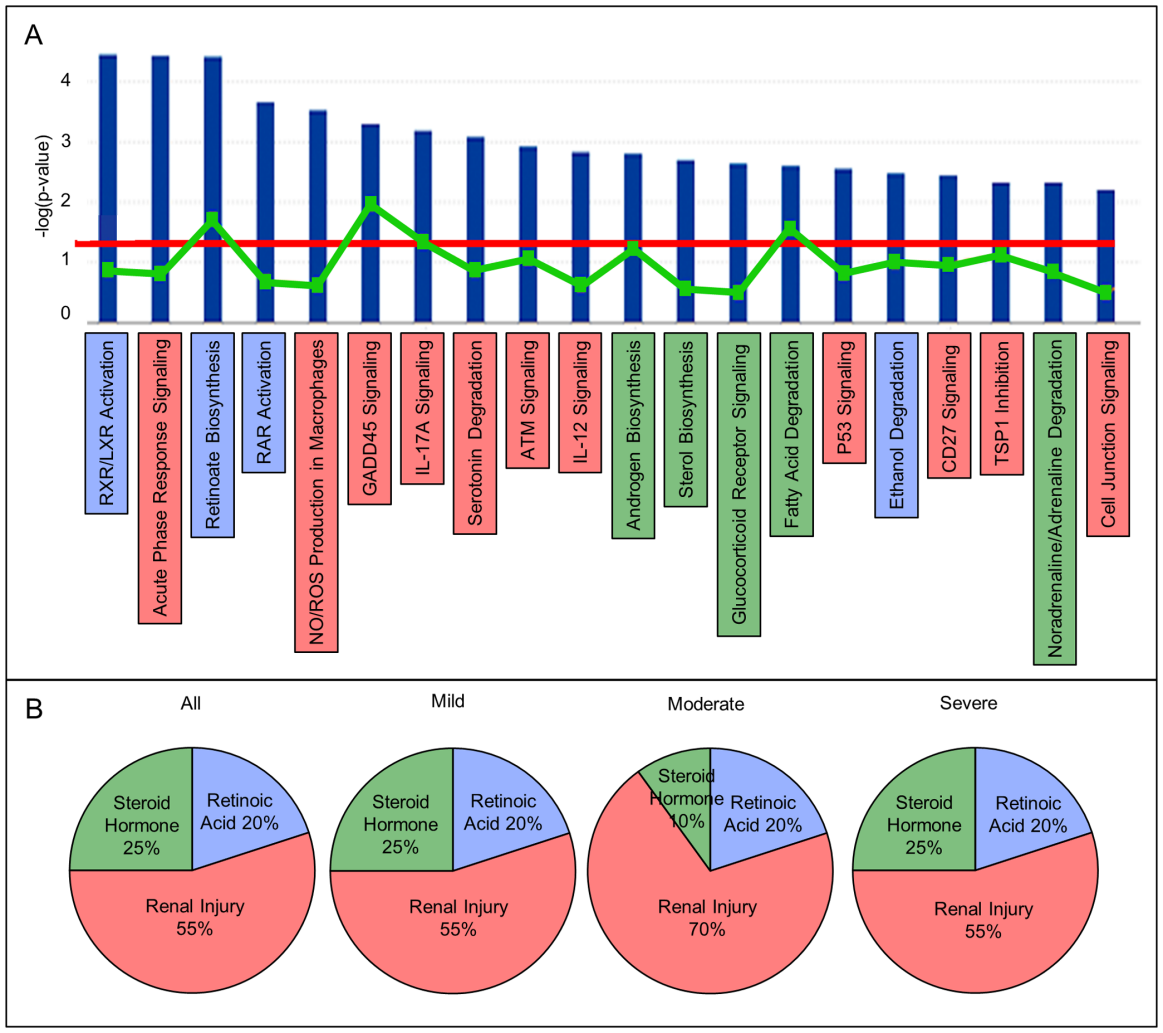
The top 20 canonical pathways in mutant versus control kidneys encompass three primary cellular processes. (**a**) Blue bars represent the -log of p-value with bar height corresponding to level of significance. The red line represents a p-value cutoff of 0.05. The green squares/line represent the ratio of the number of genes represented within each pathway that meet the cutoff criteria to the total number of genes in a given pathway. The three main cellular processes identified by IPA include retinoic acid metabolism (**blue**)**,** response to renal injury (**red**) and steroid hormone metabolism (**green**). (**b**) Canonical pathway distribution of retinoic acid metabolism (**blue**)**,** response to renal injury (**red**) and steroid hormone metabolism (**green**) in control kidneys versus all mutant, mild, moderate and severe *mgb^−/−^* kidneys.

The top five toxicological functions identified by IPA comparing *mgb^−/−^* versus control kidneys included renal necrosis/cell death, kidney failure, renal inflammation, renal nephritis and renal damage (Table S2). The top twenty canonical pathways identified by IPA encompassed three primary cellular processes: 1) response to renal injury, 2) steroid hormone metabolism and 3) retinoic acid (RA) metabolism and signaling (Figure 2A). Using qPCR we validated differential expression of 22 of 23 genes associated with retinoic acid metabolism, 17 of 21 genes associated with steroid hormone metabolism, and 21 of 43 genes associated with the response to renal injury in *mgb* ^−/−^ versus control kidneys (Figures S2–S4). The overall distribution of these cellular processes was relatively consistent at 20% RA, 25% steroid hormone and 55% renal injury pathway whether *mgb^−/−^* kidneys were compared to controls collectively or as stratified groups (Figure 2B). The top ten up and down-regulated transcripts were also associated with these same three pathways, with nine of the genes possessing overlapping functional roles in two or more (Table 1). While the overall percentage involvement of these three canonical pathways did not appear to vary between mild, moderate and severe *mgb−/−* kidneys, numerous individual gene targets showed progressive increases/decreases that correlated with the degree of hydronephrosis observed in these animals. In addition, the relative positions of the individual top twenty canonical pathways varied between mild, moderate and severe *mgb−/−* kidneys suggesting preferential utilization based upon the degree of hydronephrosis observed.

**Table 1:** Top 10 Up-Regulated and Down-Regulated Transcripts Identified by IPA of All Mutant Kidneys to Controls.

Transcript	Gene Name	Expression Value	Functions
SERPINA6	Serpin Peptidase Inhibitor, Clade A, Member 6	↑ 25.6	steroid binding/major progestin & glucocorticoid transporter
EGR1	Early Growth Response 1	↑ 11.8	DNA binding/abnormal morphology
HPD	4-Hydroxyphenol-pyruvate Dioxygenase	↑ 10.4	degradation of tyrosine
NR4A1	Nuclear Receptor Subfamily 4, Group A, Member 1	↑ 9.7	DNA binding/steroid-thyroid hormone-retinoid receptor superfamily
FOS	FBJ Murine Osteosarcoma Viral Oncogene Homolog	↑ 9.1	DNA binding/cell growth, cell death, differentiation
CYP4A14	Cytochrome P450, Family 4, Subfamily a,Polypeptide 14	↑ 7.7	arachidonic acid monooxygenase activity/hormone response
ABCC3	ATP-Binding Cassette, Subfamily 3, Member 3	↑ 7.4	ATPase activity/transporter, cell survival
EGR2	Early Growth Response 2	↑ 7.0	DNA binding/abnormal morphology
SERPINA10	Serpin Peptidase Inhibitor, Clade A, Member 10	↑ 6.9	peptidase inhibitor/proteolysis, tissue regeneration
PRLR	Prolactin Receptor	↑ 6.5	polypeptide hormone binding/anti-apoptosis; cell adhesion, epithelial differentiation, steroid biosynthesis, T-cell activation
CYP2J13	Cytochrome P450, Family 2, Subfamily j, Polypeptide 13	↓ 8.2	oxidation-reduction processes
AKR1C14	Aldo-Keto Reductase Family 1, Member C14	↓ 8.7	steroid dehydrogenase activity/androsterone, progesterone
UGT2B7	UDP Glucuronosyltransferase 2 Family,Polypeptide B7	↓ 9.5	glucuronidation in cellular damage/retinoic acid binding, steroid metabolism
CYP4A22	Cytochrome P450, Family 4, Subfamily A, Polypeptide 22	↓ 9.7	monooxygenase/cholesterol, steroid & lipid synthesis
UGT2B15	UDP Glucuronosyltransferase 2 Family, Polypeptide B15	↓ 9.8	glucuronidation in cellular damage/retinoic acid binding, steroid metabolism
CES1	Carboxyesterase 1	↓ 11.6	transesterification/hydrolysis, necrosis, retinol biosynthesis
INMT	Indolethylamin N-Methyltransferase	↓ 17.1	N- methylation/degradation
ACSM3	Acyl-CoA Synthetase Medium-Chain FamilyMember 3	↓ 29.1	fatty acid biosynthesis
SEC14l3	SEC14-Like 3	↓ 31.1	transporter activity/lipid binding
SLCO1a1	Solute Carrier Organic Anion Transporter family,Member 1a1	↓ 31.4	organic ion transporter/mediates uptake steroid conjugates/prostaglandins

The top ten activated upstream regulators included three growth factors, three intracellular signaling molecules, and four pro-inflammatory cytokines (Table 2). Transforming growth factor-beta 1 (*TGFβ1*) was the top activated upstream regulator in *mgb^−/−^* kidneys, with expression changes in 72 canonical *TGFβ1* target genes between *mgb* ^−/−^ and control kidneys (Table S3). In addition, *TGFβ3*, *Smad3* and *Smad4* were activated while *Smad7* was inhibited (Table S3). Of the 72 canonical *TGFβ1* genes, 36% represented direct targets of *Smad3/4*, while inhibitory *Smad7* was the most downregulated transcription factor identified in *mgb^−/−^* kidneys. We validated differential regulation of 13 transcriptional targets of TGFβ1 signaling between *mgb^−/−^* and control kidneys by qPCR: *Egr1*, *Fos*, *Nr4a1* (Figure S1); *Dusp1*, *Foxo3*, *Ptgs2* (Figure S3); and *Apoe*, *Cd36*, *Cdkn1a*, *Gadd45b*, *Nfkbia*, *Serpine1*, and *Stat3* (Figure S4).

**Table 2:** Top Activated Upstream Regulators Identified by IPA of All Mutant Kidneys to Controls.

Transcript (Gene Name)	z-Score	p-Value	Gene Number
TGFβ1 (transforming growth factor β-1)	5.30	8.84×10^−16^	72
IL1B (interleukin 1B)	4.42	9.93×10^−19^	54
PDGF (platelet derived growth factor)	4.40	4.46×10^−25^	42
CREB1 (cAMP responsive element binding protein 1)	4.34	1.04×10^−14^	27
TNF (tumor necrosis factor)	4.06	5.17×10^−22^	83
ERK (extracellular signal-related kinases)	3.97	1.94×10^−15^	26
NFκB (nuclear factor kappa-light-chain-enhancer of activated B cells)	3.94	4.05×10^−9^	32
IL6 (interleukin 6)	3.94	4.39×10^−12^	39
EGF (epidermal growth factor)	3.59	3.13×10^−15^	37
IFNγ (interferon gamma)	3.48	5.34×10^−7^	43

The top ten inhibited upstream regulators had roles in a wide range of cellular processes including transcription, cell death, extracellular matrix regulation, fatty acid metabolism, and immune response (Table 3). Histone deacetylase (Hdac) was the top inhibited upstream regulator identified in *mgb^−/−^* kidneys. Hdac inhibition was associated with up-regulation of 11 known target genes including six transcription factors (Table 4). We confirmed upregulation of 5 of 11 “de-repressed” Hdac target mRNAs in *mgb^−/−^* kidneys by qPCR ().

**Table 3:** Top Inhibited Upstream Regulators Identified by IPA of All Mutant Kidneys to Controls.

Transcript (Gene Name)	z-Score	p-Value	Gene Number
Hdac (histone deacetylase)	−3.24	1.62×10^−5^	11
SMAD7 (mother’s against decapentaplegic homolog 7)	−3.15	1.02×10^−6^	12
IgG (immunoglobulin G)	−3.00	1.01×10^−9^	16
ACOX1 (acetyl-CoA oxidase 1)	−2.85	3.85×10^−8^	15
SFTPA1 (surfactant protein A1)	−2.71	7.61×10^−8^	11
FOSL1 (Fos-like antigen 1)	−2.59	5.54×10^−6^	8
COL18A1 (collagen, type 18, alpha 1)	−2.20	3.27×10^−5^	9
DACH1 (dachshund homolog 1)	−2.20	5.64×10^−5^	5
TAF4 (transcription initiation factor TFIID, subunit 4)	−2.18	5.24×10^−7^	11
TNFR/FAS (tumor necrosis receptor superfamily member 6)	−1.92	3.01×10^−8^	24

**Table 4:** Hdac Target Genes Identified by IPA of All Mutant Kidneys to Controls.

Transcript (Gene Name)	Fold Change	Function
EGR1 (early growth response 1)	↑ 11.77	Transcription Factor
NR4A1 (nuclear receptor subfamily 4, group 1)	↑ 9.69	Transcription Factor
FOS (FBJ murine osteosarcoma viral oncogene homolog)	↑ 9.06	Transcription Factor
ATF3 (activating transcription factor 3)	↑ 6.34	Transcription Factor
CDKN1A (cyclin-dependent kinase inhibitor 1A)	↑ 4.36	Cyclin-Dependent Kinase Inhibitor
MT1E (metallothionein 1)	↑ 3.27	Ion Binding
JUN (jun proto-oncogene)	↑ 3.14	Transcription Factor
ARC (activity-regulated cytoskeleton-associated protein)	↑ 2.63	Actin-Binding Protein
KLF6 (Kruppel-like factor 6)	↑ 2.50	Transcription Factor
SPP1 (secreted phosphoprotein 1)	↑ 2.27	Cytokine
GADD45B (growth arrest and DNA-damage-inducible beta)	↑ 2.07	Protein Binding

The mRNA most differentially expressed between *mgb* ^−/−^ and control kidneys was *Serpina6/*corticosteroid-binding globulin (*Cbg*), a gene that displays sexually dimorphic expression [9], [10]. Eighteen additional sexually dimorphic mRNAs were identified in *mgb^−/−^* kidneys including 12 male and 6 female transcripts that were expressed at lower or higher levels, respectively (Table 5). We confirmed differential expression of 14 sexually dimorphic mRNAs by qPCR (Figure S6).

**Table 5:** Sexually Dimorphic Genes Identified by IPA of All Mutant Kidneys to Controls.

Transcript (Gene Name)	Fold Change	p-Value
**Male**		
Slco1a1 (solute carrier organic anion transporter family, member 1a1)	↓ 31.40	2.20×10^−3^
Acsm3 (acyl-CoA synthetase medium-chain family member 3)	↓ 30.28	4.93×10^−3^
Ces1f (carboxylesterase 1F)	↓ 10.22	1.23×10^−2^
Ugt2b5 (UDP glucuronosyltransferase 2 family, polypeptide B5)	↓ 9.76	9.54×10^3^
Cyp4a12b (cytochrome P450, family 4, subfamily a, polypeptide 12B)	↓ 9.74	1.28×10^−2^
Cyp4a12a (cytochrome P450, family 4, subfamily a, polypeptide 12a)	↓ 9.18	1.24×10^−2^
Ugt2b37 (UDP glucuronosyltransferase 2 family, polypeptide B37)	↓ 8.60	1.10×10^−2^
Cyp2j13 (cytochrome P450, family 2, subfamily j, polypeptide 13)	↓ 8.19	1.23×10^−2^
Cyp7b1 (cytochrome P450, family 7, subfamily b, polypeptide 1)	↓ 6.65	6.93×10^−3^
Slc7a13 (solute carrier family 7, [cationic amino acid transporter, y+ system])	↓ 6.31	2.46×10^−2^
Timd2 (T-cell immunoglobulin and mucin domain containing 2)	↓ 3.43	1.37×10^−2^
Ugt8a (UDP galactosyltransferase 8A)	↓ 2.29	2.47×10^−2^
**Female**		
Gsta2 (glutathione S-transferase, alpha 2 [Yc2])	↑ 2.67	1.30×10^−2^
Bhmt (betaine-homocysteine methyltransferase)	↑ 3.01	1.19×10^−2^
Slc7a12 (solute carrier family 7 [cationic amino acid transporter, y+ system], member 12)	↑ 3.43	5.55×10^−3^
Prlr (prolactin receptor)	↑ 6.52	6.44×10^−3^
Abcc3 (ATP-binding cassette, sub-family C [CFTR/MRP], member 3)	↑ 7.37	1.72×10^−3^
Serpina6 (serine [or cysteine] peptidase inhibitor, clade A, member 6)	↑ 25.55	9.40×10^−4^

No significant differentially expressed transcripts were identified when the individual transcriptomes of mild and moderate or moderate and severe kidneys were directly compared. In contrast, when mild and severe kidneys were compared, a single gene was expressed at ≥2-fold higher levels in mild kidneys (*Cyp2d22*) and 80 genes were expressed ≥2-fold higher levels in severe kidneys (Table S4). Interestingly, 13/80 genes including 6/10 of the most differentially expressed genes between mild and severe kidneys were expressed by renal urothelium. These included genes with roles in maintaining urothelial integrity such as uroplakins and cytokeratins, transcription factors regulating urothelial cell fate, and genes with currently unknown urothelial functions (Table 6) [11]–[15]. We confirmed expression differences in 11 urothelial mRNAs between *mgb* ^−/−^ kidneys with mild and severe hydronephrosis by qPCR (Figure S7).

**Table 6:** Urothelial Genes Identified by IPA of Mild and Severe Kidneys.

Transcript (Gene Name)	Fold Change	Function
Upk1a (uroplakin 1A)	↑ 7.29	Urothelial Integrity
Upk1b (uroplakin 1B)	↑ 8.98	Urothelial Integrity
Upk2 (uroplakin 2)	↑ 8.99	Urothelial Integrity
Upk3a (uroplakin 3A)	↑ 8.15	Urothelial Integrity
2310043J07Rik (uroplakin 3B-like)	↑ 3.95	Unknown
Krt14 (cytokeratin 14)	↑ 5.56	Urothelial Integrity
Krt18 (cytokeratin 18)	↑ 3.62	Urothelial Integrity
Krt19 (cytokeratin 19)	↑ 6.32	Urothelial Integrity
Grhl3 (grainyhead-like 3, Drosophila)	↑ 5.94	Transcription Factor
Foxa1 (forkhead box A1)	↑ 5.74	Transcription Factor
Sprr1a (small proline-rich protein 1A)	↑ 8.11	Unknown
Gjb6 (gap junction protein, beta 6, 30 kDa)	↑ 6.66	Unknown
Gsdmc4 (gasdermin C4)	↑ 5.20	Unknown

**Figure 3:**
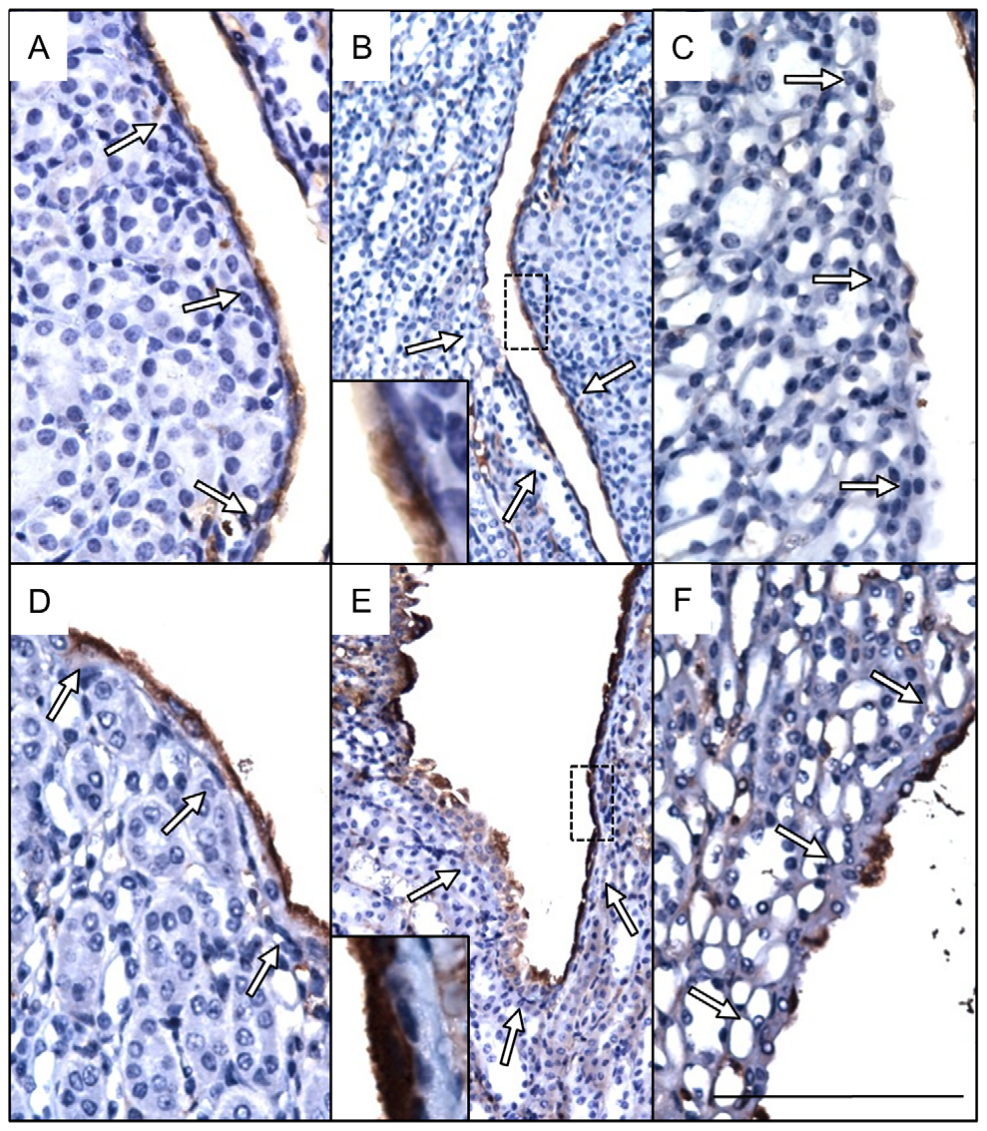
Uroplakin 3a expression and cellular distribution is altered in severe kidneys. Immunohistochemical localization (**brown, arrows**) of Upk3a shows relatively uniform expression in urothelial cells in cortical (**a, d**) and cortical-medullary (**b, e**) regions of control (**a, b**) and severe (**d, e**) kidneys. The intensity of Upk3a staining is greater within urothelial cells of severe kidneys compared to controls (**compare inset b to e**). Increased Upk3a expression was observed in urothelial cells of the medullary papillae in severe (**f**) versus control (**c**) kidneys. Scale bars are 100 microns (**a, c, d, f**), 200 microns (**b, e**) and 40 microns (**inset b, e**).

**Figure 4:**
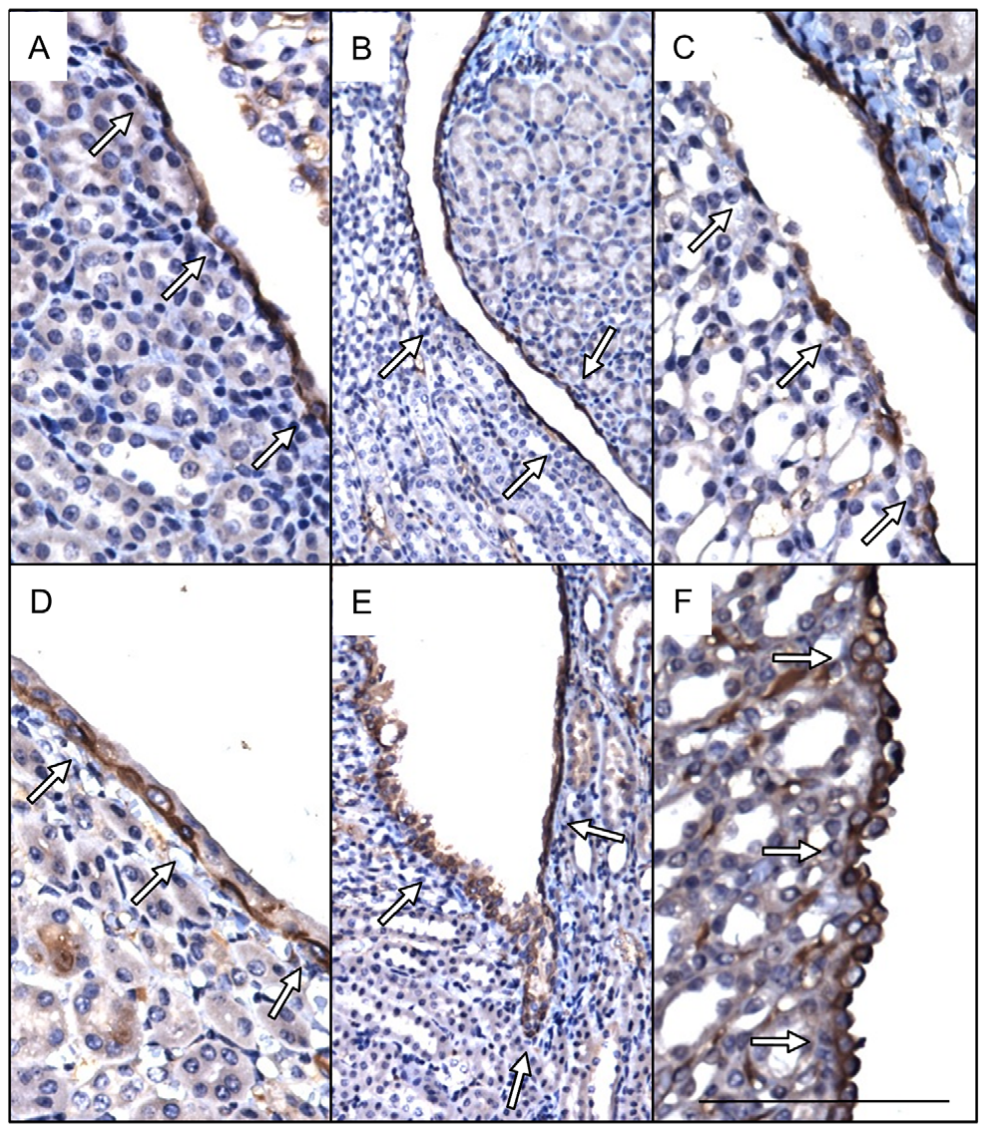
Cytokeratin 14 expression is increased along in severe kidneys. Immunohistochemical localization (**brown, arrows**) of Krt14 shows relatively uniform expression in urothelial cells in cortical (**a, d**) and cortical-medullary (**b, e**) regions of control (**a, b**) and severe (**d, e**) kidneys. Increased Krt14 expression was observed in urothelial cells of the medullary papillae in mutant (**f**) versus control (**c**) kidneys. Scale bars are 100 microns (**a, c, d**, **f**), 200 microns (**b, e**).

**Figure 5:**
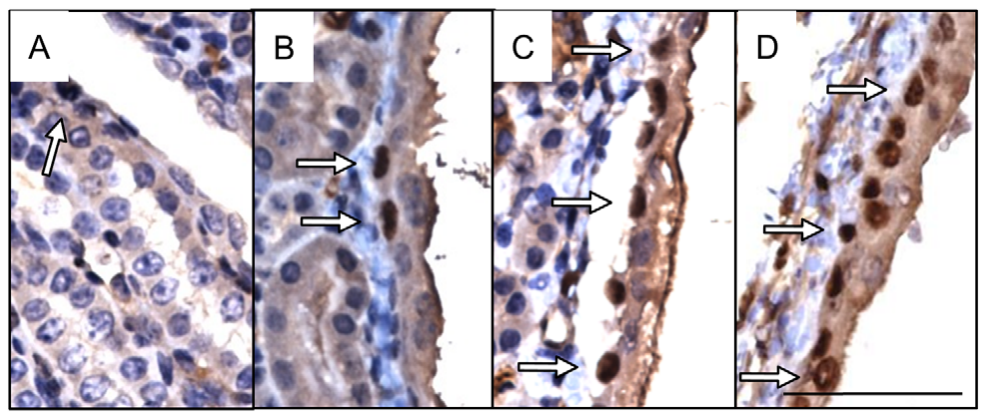
Ki-67 expression is increased in severe kidneys. Immunohistochemical localization (**brown, arrows**) of Ki-67 expression is limited to sparse individual cells within control kidney urothelium (**a**). Severe kidney urothelium shows an increase in the number of Ki-67 positive cells (**b**) including long stretches of positive cells in mutant cortical urothelium (**c**) and in mutant ureteropelvic urothelium (**d**). Scale bar is 100 microns.

Since urothelial genes appeared preferentially upregulated in *mgb* ^−/−^ kidneys, we compared urothelial morphology in severe kidneys versus controls (Figures 3–5). Control renal urothelium was principally comprised of a single layer of cuboidal cells with intermittent multilayered regions of 2–3 cells. In contrast, the renal urothelium of severe kidneys contained numerous multilayered regions of 3–5 cells throughout the renal pelvis and showed frequent cellular blebbing.

Uroplakin 3a (Upk3a), a transmembrane protein that forms apical complexes on urothelial cells, was uniformly expressed along control urothelium but showed decreased expression ascending the renal papillae resulting in only scarce individually stained cells found on the upper 2/3 of the papillae (Figure 3A–C). A similar pattern of expression was observed in the urothelium of severe kidneys except that the intensity of corticomedullary Upk3a staining appeared greater and the number of positive cells on the renal papillae appeared to increase (Figure 3D–F).

Cytokeratin 14 (Krt14), a progenitor cell marker in renal urothelium, was expressed throughout control urothelium with decreased expression as urothelial cells ascended the renal papillae (Figure 4A–C) [16]. In contrast, Krt14 expression in severe kidneys was uniformly expressed throughout the renal urothelium including the papillae where expression was significantly increased versus controls (Figure 4D–F).

**Figure 6:**
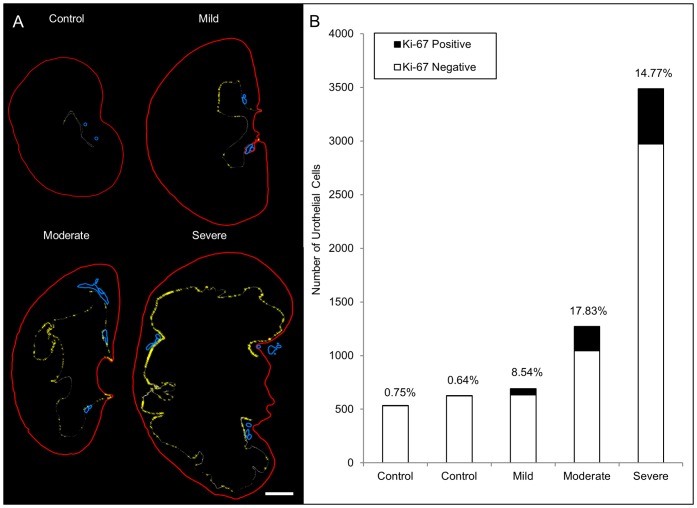
Severe kidneys show increased urothelial proliferation at the corticomedullary and ureteropelvic junctions. (**a**) Schematic reconstruction of representative control and mild, moderate and severe kidneys showing kidney capsule (**red**), renal vasculature (**blue**), Ki-67 negative renal urothelial cells (**white dots**) and Ki-67 positive renal urothelial cells (**yellow crosshairs**). Scale bar is 1000 microns. (**b**) Quantitation of the number of Ki-67 positive (**black**) versus negative (**white**) urothelial cells with percentage of Ki-67 positive urothelial cells indicated above each bar.

Cellular proliferation within the renal urothelium was assessed using Ki-67 (Figure 5) [17]. Minimal Ki-67 staining was observed in control renal urothelium with only rare individual positive cells identified. In contrast, severe kidneys showed a 20-fold increase (p≤0.02) in the percentage of Ki-67-positive cells within the renal urothelium versus controls (Figure 6). Long clusters of positive cells were observed along the corticomedullary (Figure 5C) and ureteropelvic (Figure 5D) junctions.

**Figure 7:**
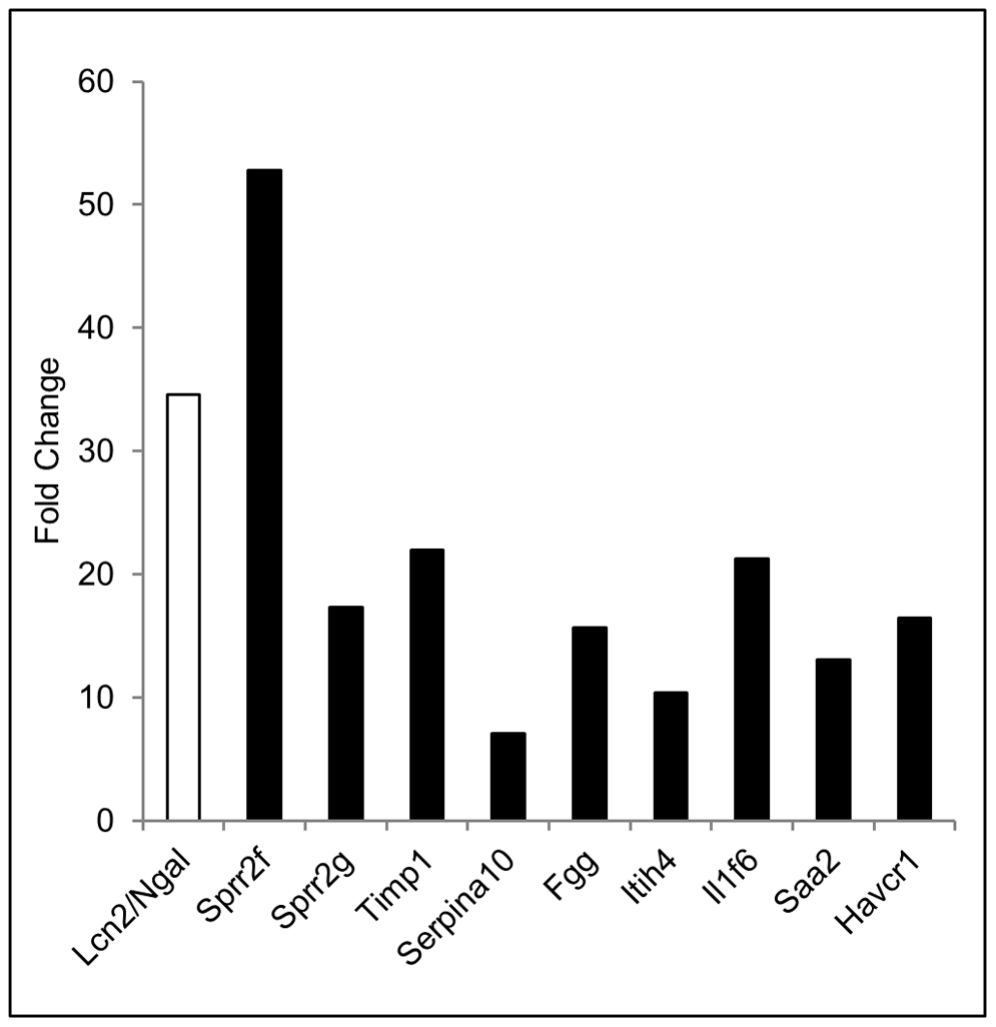
Differential expression of potential biomarkers of renal injury in *Ngal^high^* versus *Ngal^low^* kidneys. Microarray results showing fold-change in expression of potential biomarkers of renal injury comparing *Ngal^high^* versus *Ngal^low^* kidneys. (Sprr2f) small proline-rich protein 2F, Sprr2g, (Timp1) tissue inhibitor metalloproteinase 1, (Serpina10) serpin peptidase inhibitor, clade A, member 10, (Fgg) fibrinogen gamma chain, (Itih4) inter-alpha-trypsin inhibitor heavy chain family, member 4, (Il1f6) interleukin-1 family member 6, (Saa2) serum amyloid A2 and (Havcr1) hepatitis A virus cellular receptor 1.

Biomarker analysis identified a subset of three moderate and one severe hydronephrotic kidneys expressing high levels of *Ngal/Lcn2,* a well-characterized marker of tubular injury [18]–[20]. qPCR analysis validated *Ngal* expression in all 24 kidneys used in this study confirming that these four kidneys had *Ngal* mRNA expression levels at least three standard deviations higher than the mean (Figure S8). We next grouped *mgb* ^−/−^ transcriptomes with elevated *Ngal* expression (*Ngal^high^*) and analyzed for global differences in expression compared to *mgb* ^−/−^ kidneys with low *Ngal* expression (*Ngal^low^*). This analysis identified nine potential biomarkers of renal injury including several genes previously associated with tubular injury (*Havcr1*, *Il1f6*, *Fgg, Timp1*) as well as a set of novel genes not previously implicated as biomarkers of renal injury including *Sprr2f*, *Sprr2g*, *Serpina10*, *Itih4* and *Saa2* (Figure 7). We confirmed differential mRNA expression of 8 of these known or candidate tubular injury markers between *Ngal^high^* and *Ngal^low^ mgb* ^−/−^ kidneys by qPCR (Figure S9).

## Discussion

The *mgb* ^−/−^ mouse represents a unique model for the study of CON and progressive renal injury. Prior studies show that *mgb* ^−/−^ kidneys display an adaptive response to the development of progressive hydronephrosis [6]. To better understand the molecular mechanisms controlling this adaptive response, whole genome expression arrays were compared between mild, moderate and severe kidneys versus controls. This analysis suggested that the progressive development of hydronephrosis in *mgb^−/−^* kidneys principally involves three canonical pathways including: 1) renal response to injury, 2) RA pathway and 3) steroid hormone metabolism.

### Renal Injury

Over half of the top twenty canonical pathways identified in this study involved renal response to injury. This finding is consistent with prior studies showing that the progression of renal injury in *mgb^−/−^* kidneys correlates with the severity of hydronephrosis [6]. Affected kidneys showed an adaptive response that involves the appearance of α-smooth muscle actin (SMA) positive myofibroblasts, expanded TGFβ1 and CTGF expression, and the development of limited renal fibrosis [6]. This study confirms and expands those original observations at the molecular level by demonstrating that the most activated upstream regulator in *mgb* ^−/−^ kidneys is the TGFβ pathway. This observation is consistent with current literature and confirms the key role that TGFβ plays in modulating progressive renal injury and fibrosis following obstruction [21]–[23].

Although our analysis identified a group of genes that have been previously shown to be upregulated in newborn rats with UUO, it did not identify any of the key inflammatory genes previously found to be dysregulated in UUO such as interleukin-1 beta, interferon gamma regulating factor 1, and monocyte chemoattractant protein 1 [24]. Activation of the host inflammatory response is an early event in the UUO model, attributed to apoptosis of tubular epithelial cells, resulting in the release of TGFβ and reactive oxygen species which recruit and activate macrophages [25]. In contrast, apoptosis is a rare event in *mgb^−/−^* tubular epithelium, and macrophages are only detected in a subset of *mgb^−/−^* kidneys with advanced hydronephrosis [6]. We propose that the *mgb^−/−^* kidney is a low-pressure system with a more gradual onset of increased tubular hydrostatic pressure, rendering it less susceptible to tubular injury and macrophage infiltration than kidneys following UUO. The acute inflammatory response observed in certain *mgb^−/−^* kidneys with advanced hydronephrosis most likely represents a key “second hit” that worsens tubular injury resulting in expanded myofibroblast transformation/activation leading to the development of severe interstitial fibrosis. A similar “second hit” model in children with CON may be found in the form of recurrent urinary tract infections/pyelonephritis or increased back pressure resulting in ischemic injury, both of which have been associated with the subsequent development of severe interstitial fibrosis and progression to ESRD [26], [27].

Comparison of transcriptomes with varying degrees of hydronephrosis identified a urothelial gene expression signature associated with severe obstruction. Many of the urothelial genes upregulated in severe kidneys have been implicated in urothelial integrity/function, and prior studies have shown that urothelial permeability is compromised in mice deficient in *Upk2*, *Upk3a*, and *Grainyhead-like 3* [11]–[14]. The changes in Upk3a and Krt14 expression and distribution observed in severe kidneys would therefore be predicted to alter the structural integrity, permeability and functionality of the renal urothelium.

The renal urothelium also showed alterations in proliferative index and organization in severe kidneys. The corticomedullary region of proliferation identified in our study appears similar to the previously identified renal fornix, which has been proposed as a site of urothelial progenitor cells [16], [28]. These same studies showed an *Fgf7*/*Fgfr2* mediated increase in urothelial proliferation near the renal fornix in response to ischemia-reperfusion injury and UUO. *Fgf2* activation was predicted in our current analysis (p≥5.3×10^−10^) suggesting that this same signaling pathway may play a key role in renal urothelial expansion in CON.

These results suggest that urothelial cells may actively participate in the renal response to obstruction. We postulate that during the development of progressive hydronephrosis, distension of the renal pelvis results in increased proliferation of the renal urothelium and altered expression of key structural genes. Although these changes may have short-term deleterious effects on membrane permeability and function, they may be critical in initiating an adaptive, organ-protective response during pathogenic insult to the kidney. This hypothesis is consistent with our previous observation that renal fibrosis first initiates immediately beneath the renal urothelium of *mgb^−/−^* kidneys [6]. In addition to distending pelvic urothelium, obstruction leads to tubular dilatation and exposure of the entire nephron to increased hydrostatic pressure which may activate additional signaling pathways that participate in renal injury and account for some of the transcriptional changes observed in *mgb^−/−^* kidneys.

### Retinoic Acid

RA has been implicated in kidney and urinary tract development [29], [30]. We hypothesize that these same developmental functions of RA may be recapitulated during renal pathogenesis as a transient repair mechanism. Prior studies in our lab have found evidence of delayed maturation in *mgb^−/−^* kidneys including prolonged expression of early glomerular markers as well as expanded medullary expression of α-SMA [6]. RA has been shown to promote cell survival and antagonize renal fibrosis in models of glomerular injury, while administration of isotretinoin (13-cis-RA) or all*-trans* RA attenuates interstitial fibrosis and reduces TGFβ1 expression in UUO [31], [32]. In addition, administration of 13*-cis* RA to mice with ischemia-reperfusion injury results in decreased expression of the proapoptotic molecule, *Nur77*, as well as improved renal function, decreased renal injury, and reduced levels of pro-inflammatory cytokines [33]. *Nur77/Nr4a1* was among the top induced transcripts in *mgb^−/−^* kidneys suggesting a similar role for this molecule in our model system.

RA has also been shown to play a role in differentiation of urothelium [34]. This observation provides a mechanism whereby RA signaling may represent an important early urothelial response to impending renal injury. As discussed above, this hypothesis is consistent with our prior observations that the first site of collagen deposition in *mgb^−/−^* kidneys is immediately underlying the renal urothelium. In addition, the results of this study indicate that one of the earliest detectable changes in *mgb^−/−^* kidneys is an increase in renal urothelial proliferation in the face of limited pelvic distension. We postulate that these initial changes in urothelial morphology represent an early adaptive response to progressive renal injury that is designed to generate a localized and reversible repair mechanism. In the face of continued renal insult, these early remodeling/repair responses may become exacerbated and irreversible by additional contributors to renal injury resulting in permanent kidney damage.

### Steroid Hormones

The most significantly upregulated mRNA in *mgb* ^−/−^ kidneys was *Cbg*, encoding the major transport protein for glucocorticoids and progestins in the blood. *Cbg* expression in the developing and postnatal kidney is highly regulated at the mRNA and protein level [10]. While the significance of increased *Cbg* expression by *mgb* ^−/−^ kidneys remains to be determined, it seems plausible that it functions to increase the local concentration of glucocorticoid/progestin thereby downregulating the local inflammatory response. This hypothesis provides a molecular mechanism for the previous observation that *mgb* ^−/−^ kidneys display limited inflammatory infiltrates, suggesting that increased *Cbg* expression may be critical in promoting remodeling/repair following congenital obstruction by attenuating the acute inflammatory response.

In addition to altered *Cbg* levels, our study identified differential expression of genes with roles in sex hormone metabolism between *mgb* ^−/−^ and control kidneys. A variety of studies have suggested gender-based differences in the progression of renal injury [35]–[37]. Among the hypothetical mechanisms proposed for renal protection are gender-specific gene expression, gender-specific synthesis and metabolism of sex hormones, and/or modification of epigenetic factors controlling gene activation/inactivation. Interestingly, our study identified the skewed expression of a group of sexually dimorphic genes in male *mgb^−/−^* kidneys. Although the underlying mechanism(s) and significance of this observation remain to be determined, we postulate that male *mgb^−/−^* kidneys transiently alter expression of mRNAs to adopt a more “female” transcriptome under the influence of estrogen and/or other hormones. Epidemiologic data supports the concept that female gender is protective for non-diabetic renal disease progression, with higher rates of CKD progression and ESRD in males [38], [39]. Studies in animal models support a specific role for estrogen in limiting glomerulosclerosis and inflammation [36]. Tamoxifen, an estrogen partial agonist, has been shown to have anti-fibrotic effects in rats, while testosterone administration has been shown to exacerbate renal injury in the mouse UUO model [40], [41]. These observations suggest that the expression of a more “female” transcriptome in male *mgb^−/−^* kidneys may initiate a cytoprotective genetic program that transiently supports the remodeling/repair response observed in *mgb^−/−^* kidneys.

The most inhibited upstream pathway observed in *mgb^−/−^* kidneys involved Hdacs, a class of enzymes that remove acetyl groups from histones permitting tight DNA packaging that often results in the downregulation/inactivation of gene transcription [42]. Prior studies have shown that androgen, estrogen and glucocorticoid receptors are substrates/binding partners for various members of the Hdac family and that steroid hormone expression can influence perinatal programing [43]. These observations suggest that *mgb^−/−^* kidneys may reactivate Hdac-regulated genes under the modulation of local steroid hormone activity providing a potential dual role for steroids in renal pathogenesis that includes suppression of an acute inflammatory response as well as modulation of the genetic programs controlling cellular differentiation.

### Biomarkers of CON

The identification of novel biomarkers of renal injury that provide prognostic value for progression to ESRD remains a major goal in the study of kidney disease. The *mgb^−/−^* mouse model provides a unique opportunity to search for stage-specific diagnostic and prognostic markers of renal injury. Unfortunately, in this current study, we were unable to detect a single individual gene that directly correlated with our current ultrasound stratification criteria. This suggests that the degree of hydronephrosis may not always directly correlate with the molecular severity of disease progression in affected kidneys, and is consistent with clinical observations in children with CON [44]. The basis for this discordance is most likely complex, involving both genetic and epigenetic factors that may include individual genetic background, understanding of the precise chronicity of hydronephrosis/renal injury, cellular heterogeneity of whole kidney and potential loss of individual cellular phenotypes as disease severity increases.

Therefore, we sought to further stratify *mgb^−/−^* kidneys at the molecular level based upon *Ngal* expression. While *Ngal* expression was heterogeneous and did not accurately predict the degree of obstruction, a subset of moderate and severe kidneys did express significantly higher levels of *Ngal*. Assuming that this subset of *Ngal^high^* kidneys represented a consistent molecular signature associated with acute renal injury, we compared this group to the remainder of affected kidneys and controls. The detection of three previously identified markers of tubular injury validated this approach and studies are currently underway to determine the potential efficacy of the remaining five genes as stage-specific and/or prognostic markers of obstructive renal injury. Finally, identification of the renal urothelium as an early responder in kidney remodeling/repair may provide a novel target for the development of stage-specific markers of CON.

## Conclusions

**Figure 8:**
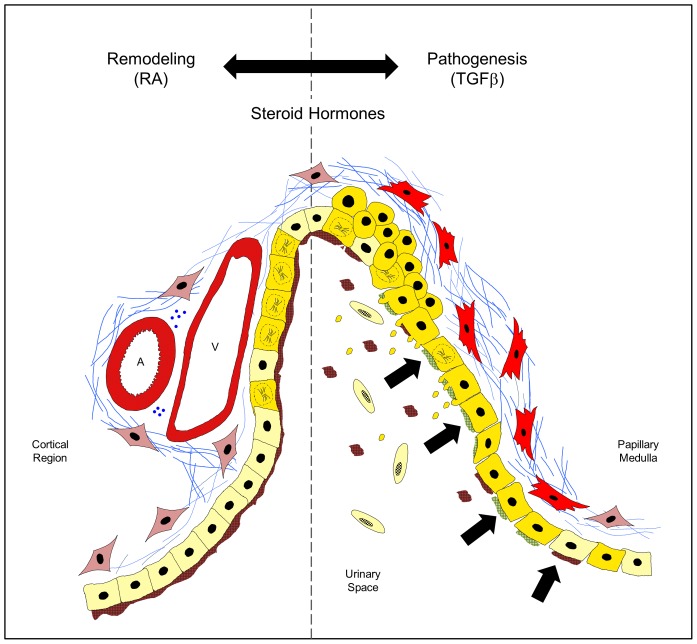
Current model of renal adaptation following the development of congenital obstruction in *mgb^−/−^* mice. Renal adaptation in *mgb^−/−^* kidneys involves a balance between tissue remodeling promoted by retinoic acid (**RA, left side of dotted line**) and renal pathogenesis promoted by transforming growth factor beta (**TGFβ, right side of dotted line**) in context of the specific steroid hormone background. Normal renal urothelium (**light yellow cells**) and submucosal fibroblasts (**light red cells**) maintain a controlled permeability barrier (**brown apical plaques**). As renal pelvic pressure increases during development of congenital obstruction (**black arrows**), the urothelium (**gold cells**) responds by dedifferentiating, increasing urothelial cellular proliferation (**mitotic nuclei**) and altering apical plaque production and composition (**green**). These changes result in urothelial blebbing and loss to the renal space, breakdown of membrane permeability, activation of submucosal myofibroblast (**red cells**) and increased deposition of extracellular matrix including collagen (**blue lines**). Neurovascular beds are indicated by: A (artery), V (vein), and blue dots (neural bundles).

The results of this study indicate that the development of progressive hydronephrosis in *mgb* ^−/−^ mice results in a highly orchestrated adaptive response in the chronically affected kidney (Figure 8). We propose that renal adaptation involves a balance between RA-initiated remodeling/repair and TGFβ directed pathogenesis. In this model, the renal urothelium plays a key role in sensing and transmitting the early signals responsible for initiating the remodeling/repair mechanisms observed in *mgb* ^−/−^ kidneys. We postulate that increasing back-pressure within the renal pelvis causes the gradual expansion of the renal urothelium from morphologically well-defined regions of proliferation. Cellular proliferation results in significant phenotypic alterations within the renal urothelium changing the composition of apical plaques and altering the functionality and permeability of the urothelial membrane. These urothelial changes most likely initiate the activation of submucosal myofibroblasts resulting in collagen deposition immediately underlying the renal urothelium – the first major histopathological finding observed in *mgb* ^−/−^ kidneys (6). Steroid hormones may play a dual role in the remodeling/repair response by directly suppressing acute inflammation and modulating the transcriptional control of gene expression thereby providing a potential mechanism for gender-based differences in renal pathogenesis and progression.

The distribution of pathways associated with RA signaling, steroid hormone metabolism, and renal injury did not vary between mildly and severely hydronephrotic *mgb^−/−^* kidneys. Nonetheless, we propose that these pathways modulate proliferation and remodeling of the renal urothelium and activation of submucosal myofibroblasts, processes that occur in all *mgb^−/−^* kidneys (Figure 6) [6]. We further postulate that these pathways may act both in a reparative fashion and to render the kidney susceptible to future pathologic changes including interstitial fibrosis and tubular injury under the influence of TGFβ, and that steroid hormones may serve a role in modulating this process (Figure 8).

Progression of renal injury in our model may be exacerbated by a “second hit” in the affected kidney. In *mgb* ^−/−^ mice, this “second hit” may occur through several processes including the development of urolithiasis, ascending infection and pyelonephritis, and/or acute high-pressure obstruction. Each of these events drives the molecular balance away from renal remodeling/repair towards expanded renal pathogenesis. Under the influence of TGFβ, increased activation of myofibroblasts leads to severe interstitial fibrosis that results in further loss of renal tubules and glomeruli and reduced renal function. It is intriguing to postulate that the structural changes induced during RA or steroid hormone mediated remodeling/repair alter the functionality of the renal urothelium increasing the kidney’s susceptibility to a “second hit” and the subsequent development of ESRD. Future studies will focus on the refinement of our current model including evaluation of the relationship between urothelial proliferation/integrity and renal injury pathways. Elucidation of these pathogenic mechanisms will lead to identification of biomarkers for risk stratification and development of therapeutic strategies designed to reduce the impact of CON.

## Supporting Information

Figure S1 **qPCR validation of a subset of differentially expressed genes in all mutant versus control kidneys.** (a) mRNA expression of the six most increased and (b) three most decreased genes in *mgb^−/−^* kidneys versus control.
(TIF)

Figure S2 **qPCR validation of a subset of differentially expressed genes implicated in retinoic acid metabolism.** Fold change of mRNA expression of 22 genes in *mgb^−/−^* (mutant) kidneys versus controls. *p = <0.05, **p = <0.005, ***p = 0.0005.
(TIF)

Figure S3 **qPCR validation of a subset of differentially expressed genes implicated in steroid hormone metabolism.** Fold change of mRNA expression of 17 genes in *mgb^−/−^* (mutant) kidneys versus controls. *p = <0.05, **p = <0.005, ***p = 0.0005.
(TIF)

Figure S4 **qPCR validation of a subset of differentially expressed genes implicated in the response to renal injury.** Fold change of mRNA expression of 21 genes in *mgb^−/−^* (mutant) kidneys versus controls. *p = <0.05, **p = <0.005, ***p = 0.0005.
(TIF)

Figure S5 **qPCR validation of a subset of differentially expressed Hdac target genes.** Fold change of mRNA expression of 5 genes in *mgb^−/−^* (mutant) kidneys versus controls. *p = <0.05, ***p = 0.0005.
(TIF)

Figure S6 **qPCR validation of a subset of differentially expressed sexually dimorphic genes.** Fold change of mRNA expression of 14 genes in *mgb^−/−^* (mutant) kidneys versus controls. *p = <0.05, **p = <0.005, ***p = 0.0005.
(TIF)

Figure S7 **qPCR validation of a subset of urothelium specific genes between mild and severe kidneys.** Fold change of mRNA expression of 11 genes in severe *mgb^−/−^* kidneys versus mild *mgb^−/−^*. *p = <0.05, **p = <0.005, ***p = 0.0005.
(TIF)

Figure S8 **qPCR validation of** ***Ngal*** **expression in mutant and control kidneys.** The average fold change of the four Ngal^high^ mutant kidneys, all Ngal^low^ (Ngal^low^ control+Ngal^low^ mutant kidneys), mutant Ngal^low^ and control Ngal^low^ kidneys compared to a control pool of kidneys. *p = 0.018, **p = 0.019 and ***p = 0.017.
(TIF)

Figure S9 **qPCR validation of potential biomarkers of renal injury.** Fold change of mRNA expression of 8 genes in Ngal^high^ kidneys versus Ngal^low^ *mgb^−/−^* kidneys. *p = <0.05, **p = <0.005, ***p = 0.0005.
(TIF)

Table S1 **Genes with ≥2-fold differential expression between all mgb−/− and wildtype kidneys.**
(XLS)

Table S2 **Top Toxicological Functions Identified by IPA of All Mutant Kidneys to Controls.**
(DOC)

Table S3 **Differentially Expressed TGF-β/Smad Target Genes Comparing All Mutant and Control Kidneys.**
(XLS)

Table S4 **Differential Gene Expression Between Mild and Severe Hydronephrotic mgb−/− Kidneys.**
(XLS)
