# Supplementary figures and images for: Molecular Basis of Renal Adaptation in a Murine Model of Congenital Obstructive Nephropathy
Source: PLoS One. 2013 Sep 4;8(9):e72762. doi: 10.1371/journal.pone.0072762 (PMC3762787; doi:10.1371/journal.pone.0072762)

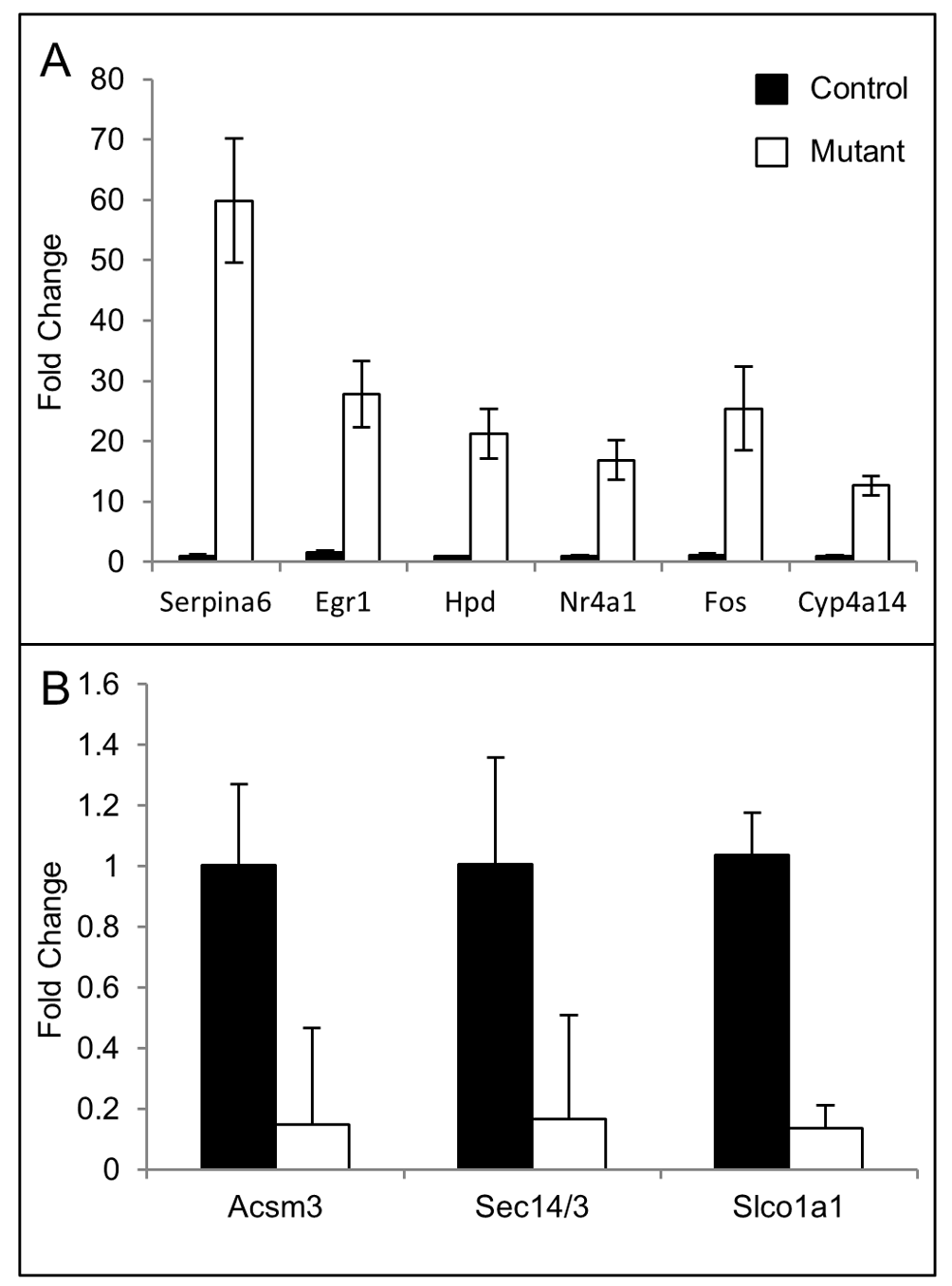

Supplement: Figure S1 — qPCR validation of a subset of differentially expressed genes in all mutant versus control kidneys. (a) mRNA expression of the six most increased and (b) three most decreased genes in mgb−/− kidneys versus control. (TIF) [file pone.0072762.s001.tif]

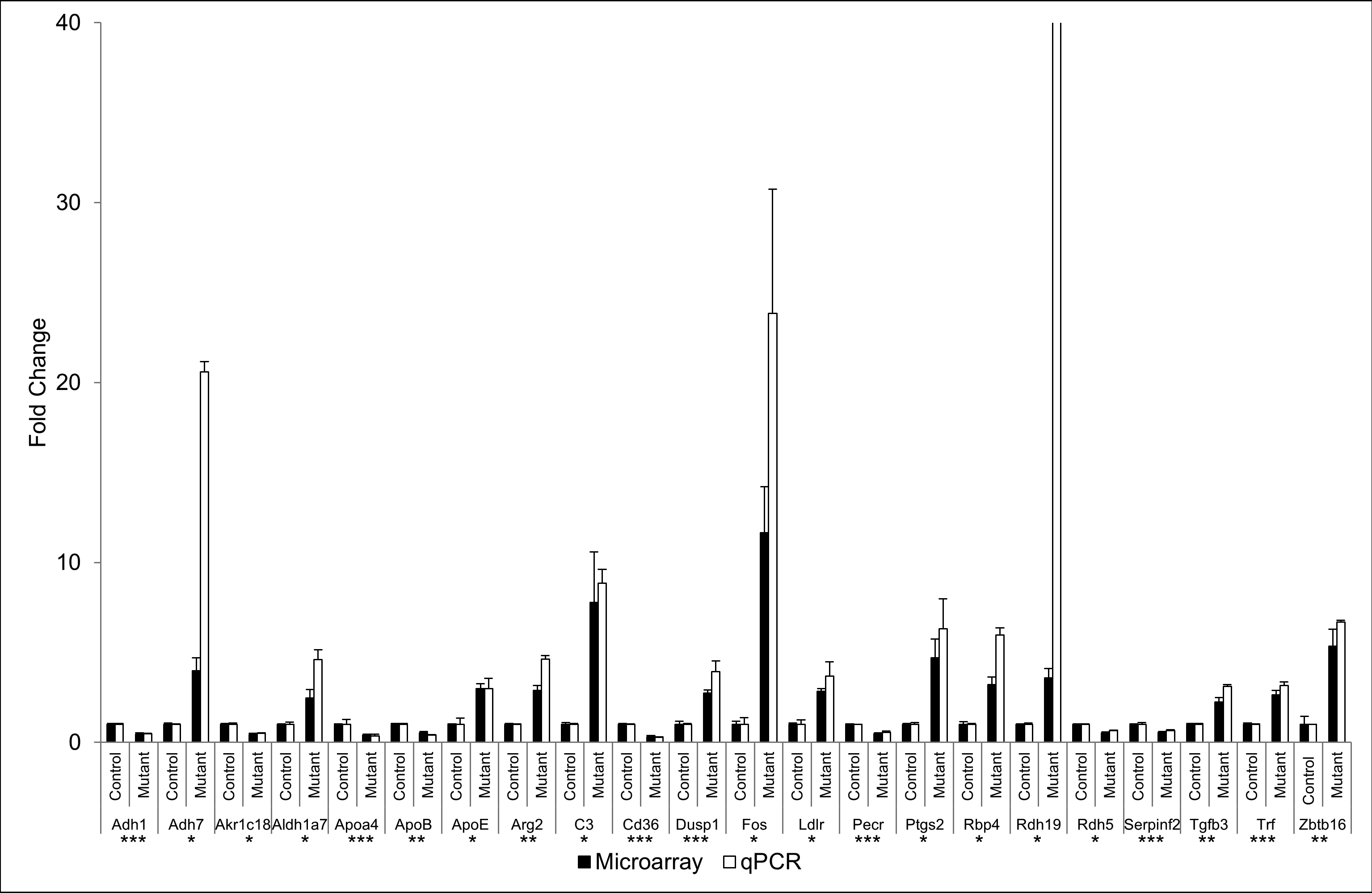

Supplement: Figure S2 — qPCR validation of a subset of differentially expressed genes implicated in retinoic acid metabolism. Fold change of mRNA expression of 22 genes in mgb−/− (mutant) kidneys versus controls. *p = <0.05, **p = <0.005, ***p = 0.0005. (TIF) [file pone.0072762.s002.tif]

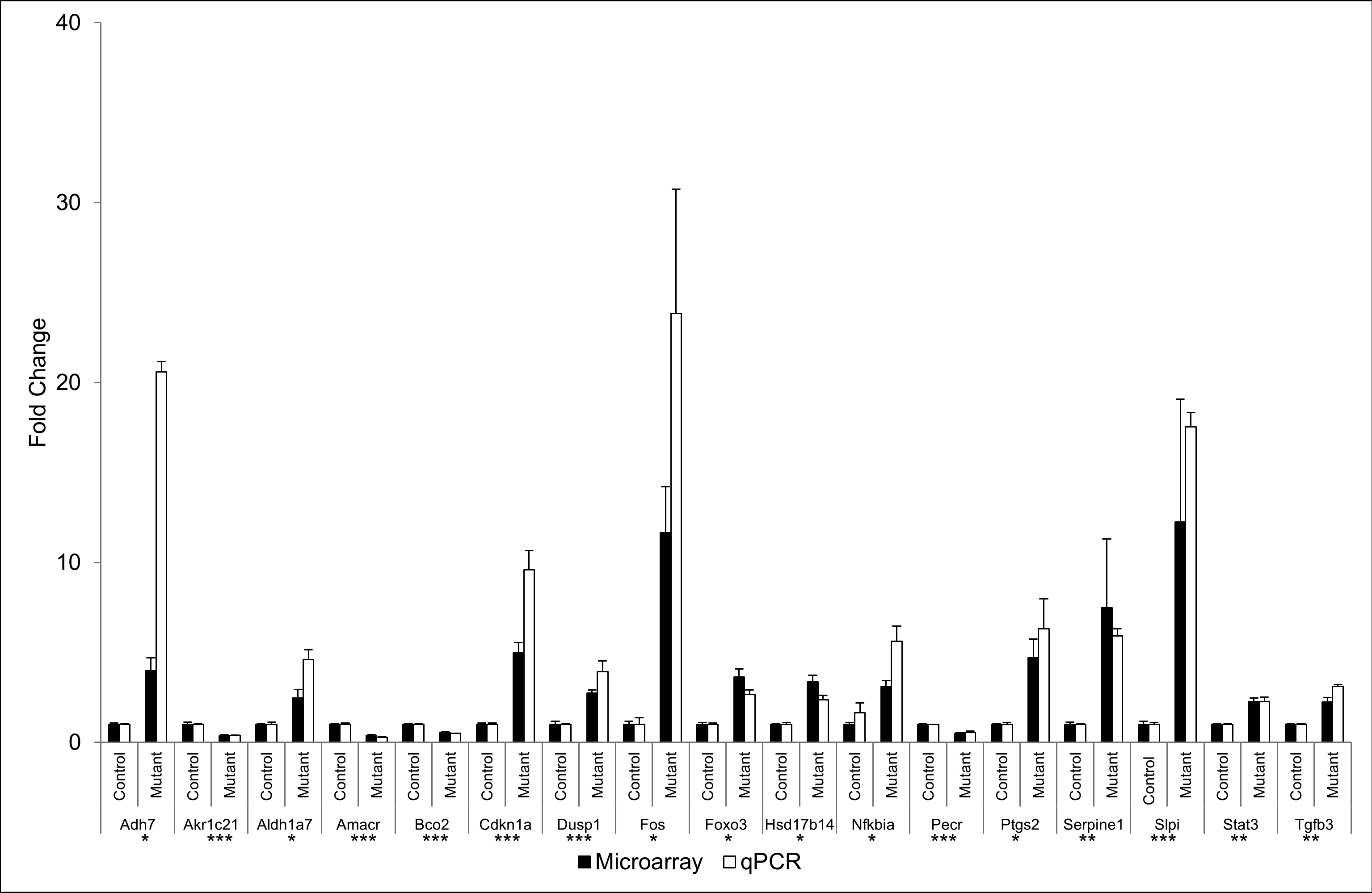

Supplement: Figure S3 — qPCR validation of a subset of differentially expressed genes implicated in steroid hormone metabolism. Fold change of mRNA expression of 17 genes in mgb−/− (mutant) kidneys versus controls. *p = <0.05, **p = <0.005, ***p = 0.0005. (TIF) [file pone.0072762.s003.tif]

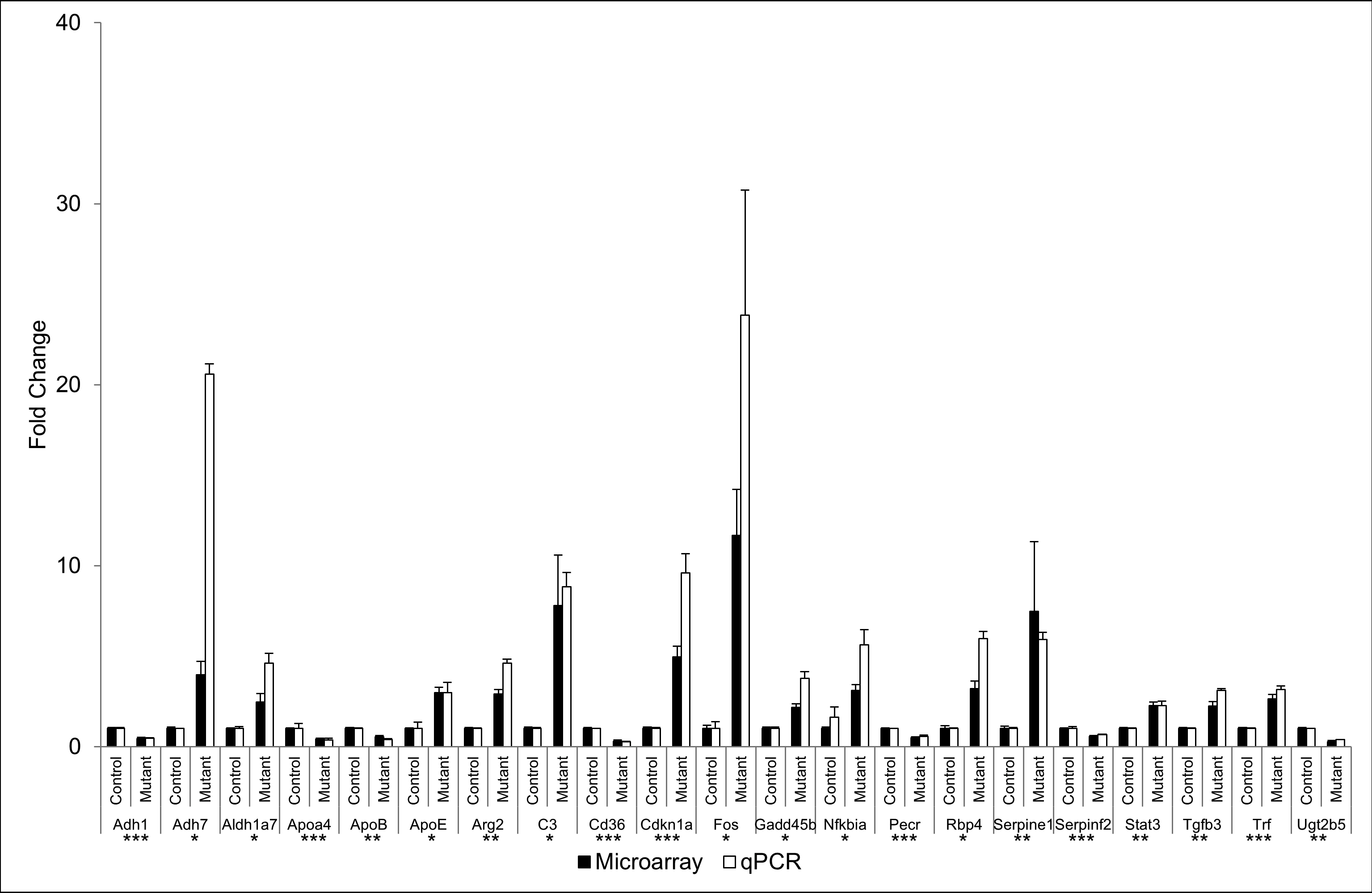

Supplement: Figure S4 — qPCR validation of a subset of differentially expressed genes implicated in the response to renal injury. Fold change of mRNA expression of 21 genes in mgb−/− (mutant) kidneys versus controls. *p = <0.05, **p = <0.005, ***p = 0.0005. (TIF) [file pone.0072762.s004.tif]

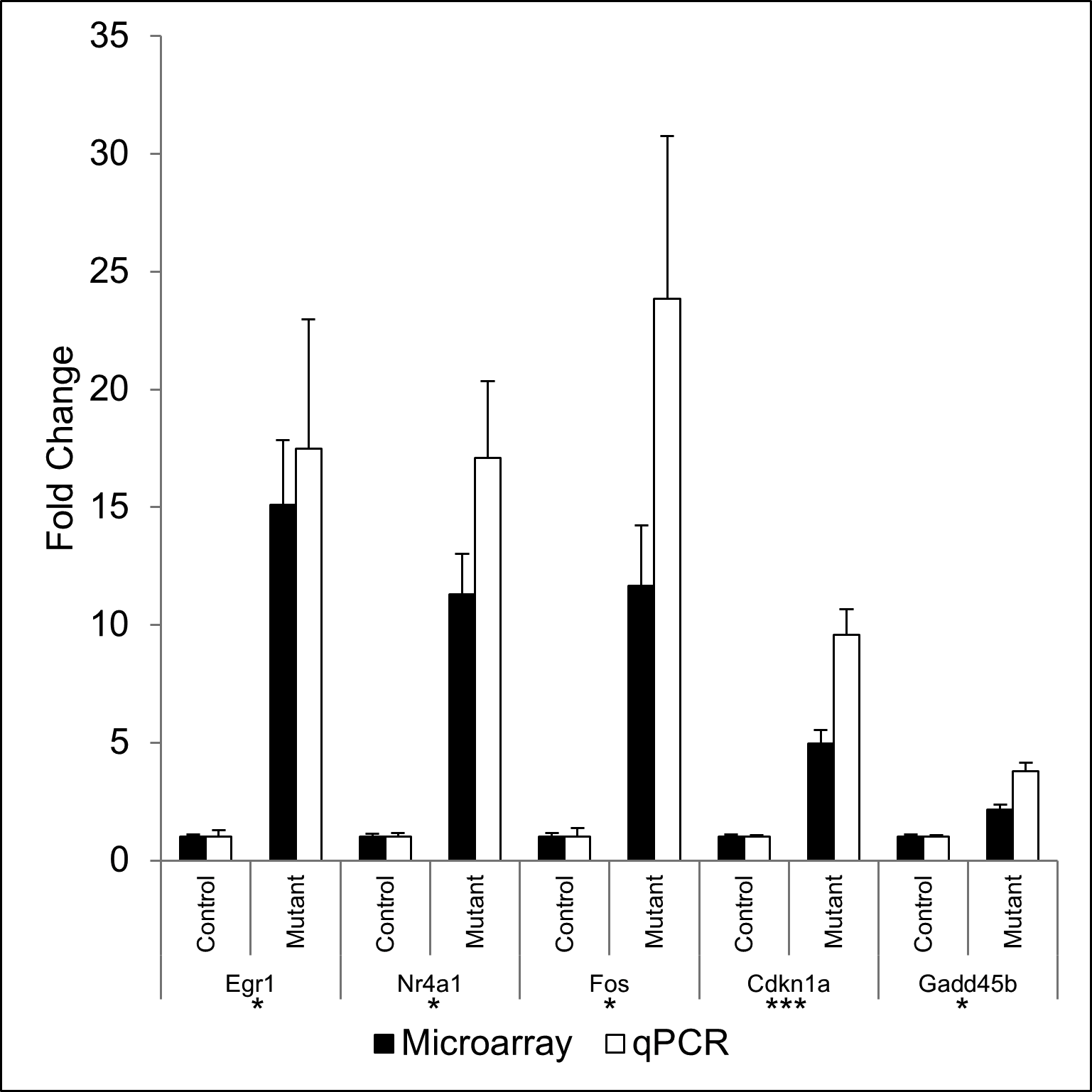

Supplement: Figure S5 — qPCR validation of a subset of differentially expressed Hdac target genes. Fold change of mRNA expression of 5 genes in mgb−/− (mutant) kidneys versus controls. *p = <0.05, ***p = 0.0005. (TIF) [file pone.0072762.s005.tif]

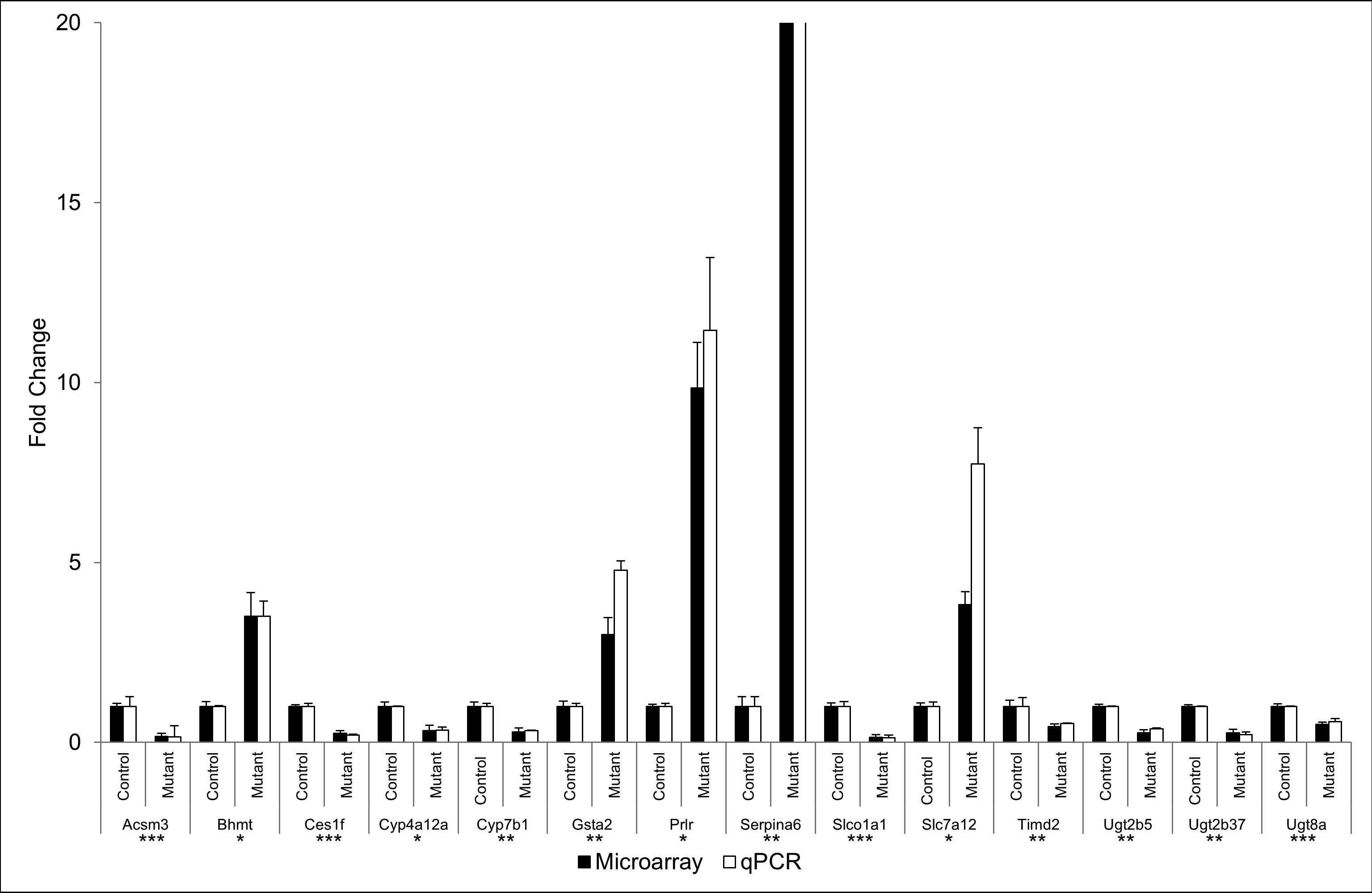

Supplement: Figure S6 — qPCR validation of a subset of differentially expressed sexually dimorphic genes. Fold change of mRNA expression of 14 genes in mgb−/− (mutant) kidneys versus controls. *p = <0.05, **p = <0.005, ***p = 0.0005. (TIF) [file pone.0072762.s006.tif]

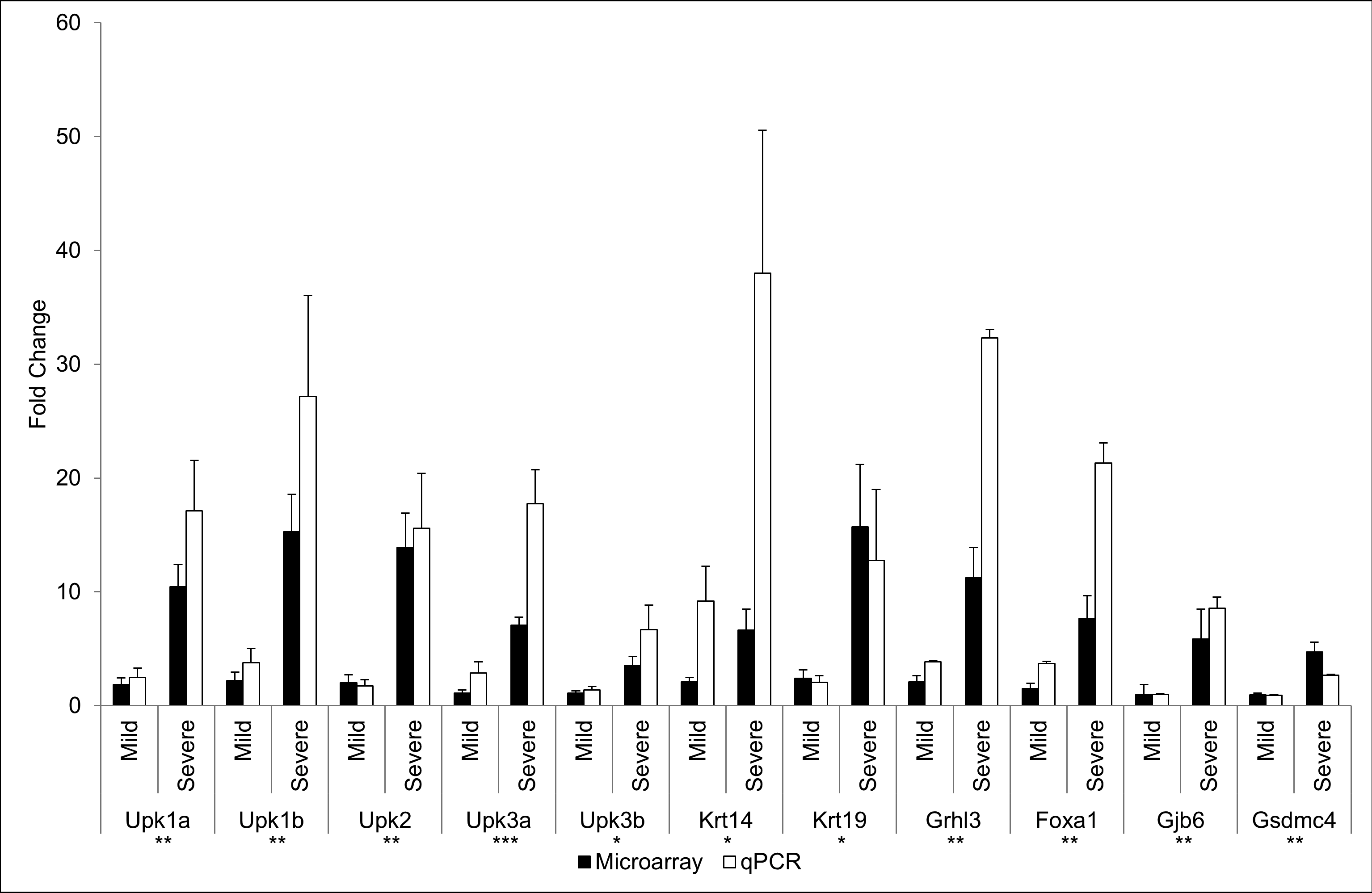

Supplement: Figure S7 — qPCR validation of a subset of urothelium specific genes between mild and severe kidneys. Fold change of mRNA expression of 11 genes in severe mgb−/− kidneys versus mild mgb−/−. *p = <0.05, **p = <0.005, ***p = 0.0005. (TIF) [file pone.0072762.s007.tif]

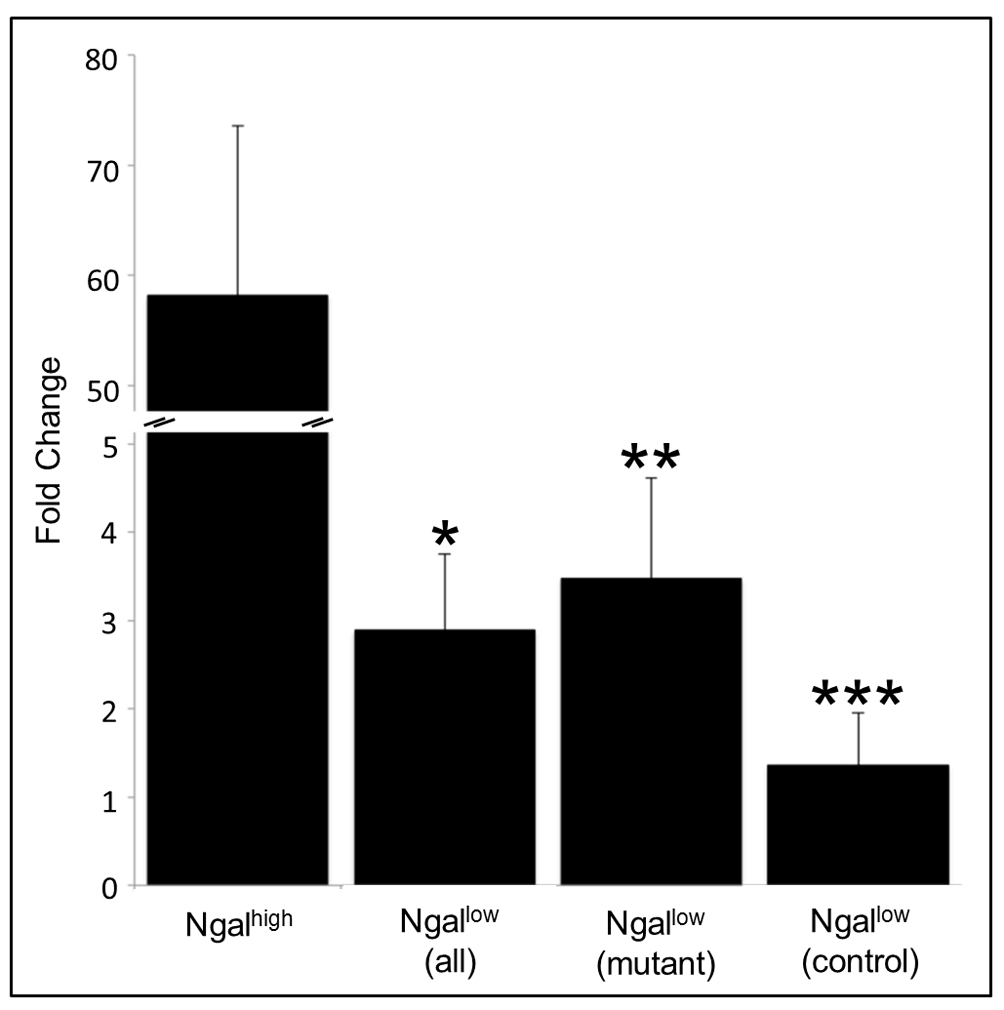

Supplement: Figure S8 — qPCR validation of Ngal expression in mutant and control kidneys. The average fold change of the four Ngalhigh mutant kidneys, all Ngallow (Ngallow control+Ngallow mutant kidneys), mutant Ngallow and control Ngallow kidneys compared to a control pool of kidneys. *p = 0.018, **p = 0.019 and ***p = 0.017. (TIF) [file pone.0072762.s008.tif]

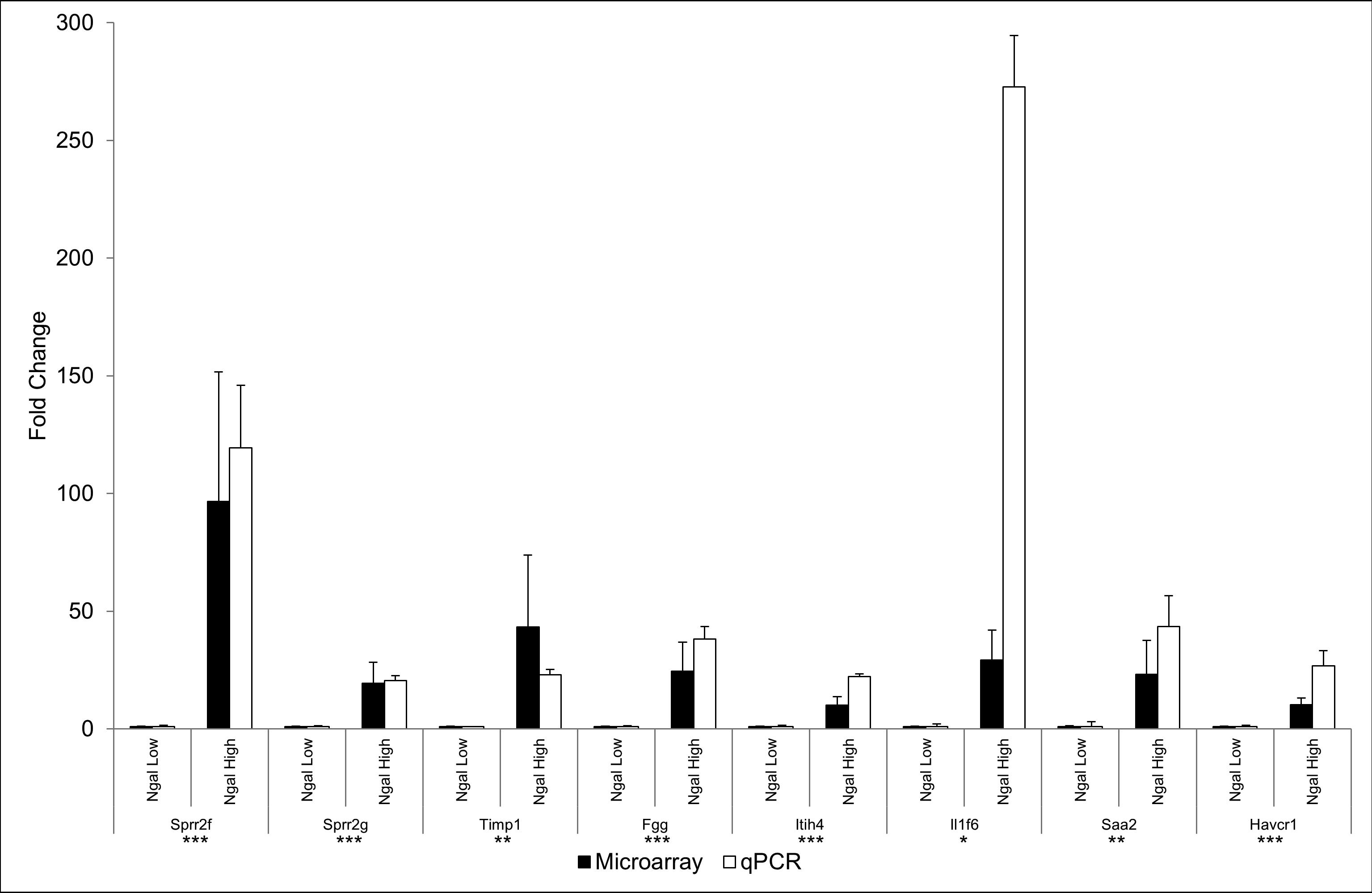

Supplement: Figure S9 — qPCR validation of potential biomarkers of renal injury. Fold change of mRNA expression of 8 genes in Ngalhigh kidneys versus Ngallow mgb−/− kidneys. *p = <0.05, **p = <0.005, ***p = 0.0005. (TIF) [file pone.0072762.s009.tif]
