# Supplementary material for: Molecular Basis of Renal Adaptation in a Murine Model of Congenital Obstructive Nephropathy
Source: PLoS One. 2013 Sep 4;8(9):e72762. doi: 10.1371/journal.pone.0072762 (PMC3762787; doi:10.1371/journal.pone.0072762)
Supplement: Table S2 — Top Toxicological Functions Identified by IPA of All Mutant Kidneys to Controls. (DOC) [file pone.0072762.s011.doc]

| **Table S2. Top Toxicological Functions Identified by IPA of All Mutant Kidneys to Controls** | | |
| --- | --- | --- |
| **Functions** | **p-Value Range** | **Gene Number** |
| Renal Necrosis/Cell Death | 8.51 X 10^-6^ – 3.23 X 10^-1^ | 20 |
| Kidney Failure | 1.90 X 10^-4^ – 1.86 X 10^-1^ | 13 |
| Renal Inflammation | 1.53 X 10^-3^ – 2.95 X 10^-1^ | 14 |
| Renal Nephritis | 1.53 X 10^-3^ – 2.95 X 10^-1^ | 14 |
| Renal Damage | 2.04 X 10^-3^ – 1.50 X 10^-1^ | 15 |
